# Tuning of Cationic Polymer Functionality in Complex Coacervate Artificial Cells for Optimized Enzyme Activity

**DOI:** 10.1021/acs.biomac.3c01063

**Published:** 2023-12-08

**Authors:** Alexander B Cook, Bruno Delgado Gonzalez, Jan C M van Hest

**Affiliations:** † Bio-Organic Chemistry, Institute for Complex Molecular Systems, Eindhoven University of Technology, Eindhoven 5600 MB, Netherlands; ‡ Departamento de Química Orgánica, Centro Singular de Investigación en Química Biolóxica e Materiais Moleculares (CIQUS), Universidade de Santiago de Compostela, Jenaro de la Fuente s/n, Santiago de Compostela 15782, Spain; § Biomedical Engineering, Institute for Complex Molecular Systems, Eindhoven University of Technology, Eindhoven 5600 MB, Netherlands

## Abstract

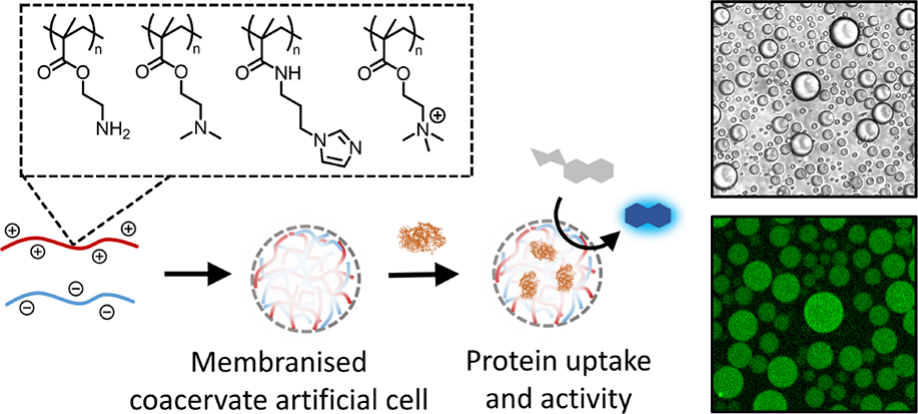

Complex coacervates are a versatile platform to mimic the structure of living cells. In both living systems and artificial cells, a macromolecularly crowded condensate phase has been shown to be able to modulate enzyme activity. Yet, how enzyme activity is affected by interactions (particularly with cationic charges) inside coacervates is not well studied. Here, we synthesized a series of amino-functional polymers to investigate the effect of the type of amine and charge density on coacervate formation, stability, protein partitioning, and enzyme function. The polymers were prepared by RAFT polymerization using as monomers aminoethyl methacrylate (AEAM), 2-(dimethylamino)ethyl methacrylate (DMAEMA), imidazolepropyl methacrylamide (IPMAm), and [2-(methacryloyloxy)ethyl] trimethylammonium chloride (TMAEMA). Membranized complex coacervate artificial cells were formed with these polycations and an anionic amylose derivative. Results show that polycations with reduced charge density result in higher protein mobility in the condensates and also higher enzyme activity. Insights described here could help guide the use of coacervate artificial cells in applications such as sensing, catalysis, and therapeutic formulations.

## Introduction

Living organisms have evolved from complex mixtures of proteins, salts, nucleic acids, lipids, and many other molecules, to be advanced self-sustaining and interactive entities.^1^ Both liquid–liquid phase separation and compartmentalization are seen to be key to this evolution, as they can help regulate complex biochemical reactions in a spatiotemporal manner.

Artificial cell research is beginning to uncover new insights in how complex natural cells function, by combining the fields of synthetic biology, macromolecular chemistry, and nanotechnology to engineer cell-like environments with functional bio molecular components.^2^ For example, polymer conjugates and lipid-based giant unilamellar vesicles have been employed to create cell-like compartments able to carry out protein translation,^3,4^ enzymatic cascades,^5^ or DNA network reactions.^6−8^ Water-in-oil droplets and soft hydrogel microparticles, both membrane-bound and membrane free, have also shown to be promising systems to mimic cell behavior.^9−11^ An interesting class of artificial cells is based on complex coacervates, which have been compared to prebiological reactive compartments since the early 1900s.^12^ Complex coacervates are formed when charged molecules, and to a larger extent macromolecules, spontaneously form liquid condensates through associative processes. In associative systems, the macromolecularly crowded and usually charged liquidlike environment can more accurately mimic the complex nature of the cell cytoplasm. Biomolecules such as RNA and proteins significantly accumulate inside coacervate droplets compared with the dilute phase. This phenomenon has led to rate enhancements for enzymes and catalytic RNA reactions,^13,14^ as well as single-stranded–double-stranded RNA duplex formation kinetics.^15^

The structure of the condensate forming polymers has recently been shown to play an important role in preserving the function of proteins encapsulated in this phase.^16,17^ Local protein surfaces are heterogeneous and have many chemical functionality variations. Interactions of macromolecular species with proteins and monomer–amino acid interactions can have an influence on protein folding.^18^ Low-multivalency coacervate-forming charged polypeptides impart higher ribozyme activity compared to longer polymers of arginine and lysine functionality.^19^ Furthermore, incorporation of neutral monomers into the polymer microstructure to reduce charge density also increases ribozyme activity in polypeptide coacervates.^20^ Xu et al. developed a synthetic heteropolymer composed of methyl methacrylate (MMA), oligo (ethylene glycol) methacrylate (OEGMA), and 2-ethylhexyl methacrylate (EHMA), with the addition of one of the following charged monomers 3-sulfopropyl methacrylate potassium salt (SPMA), or 2-(dimethylamino) ethyl methacrylate (DMAEMA). Protein function was preserved by variation of monomer ratios, sequences, and block lengths, as well as the presence of one of the charged monomers.^17^ By optimizing these polymer structure parameters, the authors showed many benefits including assistance of protein folding during translation, enhanced thermal stability, and cytosol mimicking properties; however, the type of amine employed was not varied from the tertiary amine in DMAEMA.

The importance of chemical structure for the function of macromolecular condensates is clear, and while most of these studies involve cationic species, there has been little investigation into the role of amine functionality and charge density in protein activity. Here, we use synthetic coacervates to tune the charged nature of polyelectrolytes in complex coacervate droplets. A range of midlength polymer structures with different amine substituent monomers were synthesized to investigate how cationic charge structure affects protein activity in artificial cells. Primary amine, dimethyl tertiary amine, imidazole base amine, and trimethyl quaternary amine-based monomers were polymerized by RAFT polymerization, and complex coacervate artificial cells were formed with carboxymethyl amylose. The coacervates were stabilized with a terpolymer membrane. The physical properties, salt stability, and size characteristics of the coacervates were studied. Protein uptake and condensate mobility were determined using confocal microscopy and FRAP, and finally, the enzyme activity of β-galactosidase in artificial cells formed from different polycations was determined with fluorescence assays. The role of the amine structure was found to play a decisive role in condensate activity, which we hypothesize to be due to stronger ion pair interactions and reduced protein mobility.

## Experimental Section

### Materials

Methacryloyl chloride, 1-(3-aminopropyl)imidazole, triethyl amine, 4,4-azobis(4-cyanovaleric acid), (2-Boc-amino)ethyl methacrylate, 2-(dimethylamino)ethyl methacrylate, and [2-(methacryloyloxy)ethyl] trimethylammonium chloride solution (75 wt % in water) were purchased from Merck KGaA. The RAFT polymerization chain transfer agents 4-cyano-4-[(ethylsulfanylthiocarbonyl)sulfanyl] pentanoic acid and methyl 4-cyano-4-(dodecylthiocarbonothioylthio)pentanoate were purchased from ABCR GmbH. Succinic acid and AF488 dye dual-modified bovine serum albumin (succ-BSA-488), carboxymethyl amylose, and terpolymer mPEG-p(CL-*g*-TMC)-pGlu were prepared and purified as previously reported.^21^ The terpolymer synthesis is described in the SI. For enzyme assays, β-galactosidase, from *Escherichia coli*, lyophilized powder, ≥500 units/mg protein (β-Gal), 4-methylumbelliferyl β-d-galactopyranoside (4-MUG), and 4-methylumbelliferone, were purchased from Merck KGaA. All other chemicals and solvents were purchased from Merck KGaA.

### Synthesis of 3-(Imidazole)propyl Methacrylamide (IPMAm)

To a 100 mL double-neck round-bottom flask equipped with a magnetic stir bar were added 1-(3-aminopropyl)imidazole (6.39 mmol, 0.760 mL), dichloromethane (40 mL), and triethylamine (8.53 mmol, 1.20 mL) under a nitrogen atmosphere, and the reaction mixture was cooled to 0 °C. Methacryloyl chloride (6.09 mmol, 0.593 mL) in dichloromethane (5 mL) was subsequently added dropwise over an hour with stirring. The reaction proceeded at room temperature for approximately 16 h with continued stirring, after the addition of methacryloyl chloride. Upon completion, the reaction mixture was washed first with water (3×) and last with brine. The organic layer was dried over anhydrous MgSO_4_ and filtered, and the solvent was removed via rotary evaporation. The product was purified by flash column chromatography (ethyl acetate/methanol, 95/5) to give imidazolepropyl methacrylamide as a clear colorless oily liquid. Structure and purity were confirmed by ^1^H NMR spectroscopy (Figure S6). ^1^H NMR (400 MHz, CDCl_3_): δ 7.47 (s, 1H, N–C**H**=N), 7.04 (s, 1H, N–C**H**=CH), 6.95 (s, 1H, CH=C**H**–N), 5.67 (s, 1H, C**H**_2_=C); 5.33 (s, 1H, C**H**_2_=C), 4.00 (t, 2H, CH_2_–C**H**_2_–N), 3.34 (m, 2H, NH–C**H**_2_–CH_2_), 2.04 (m, 2H, CH_2_–C**H**_2_–CH_2_), 1.94 (s, 3H, C**H**_3_–C) ppm.

### RAFT Polymerizations

**Figure 1:**
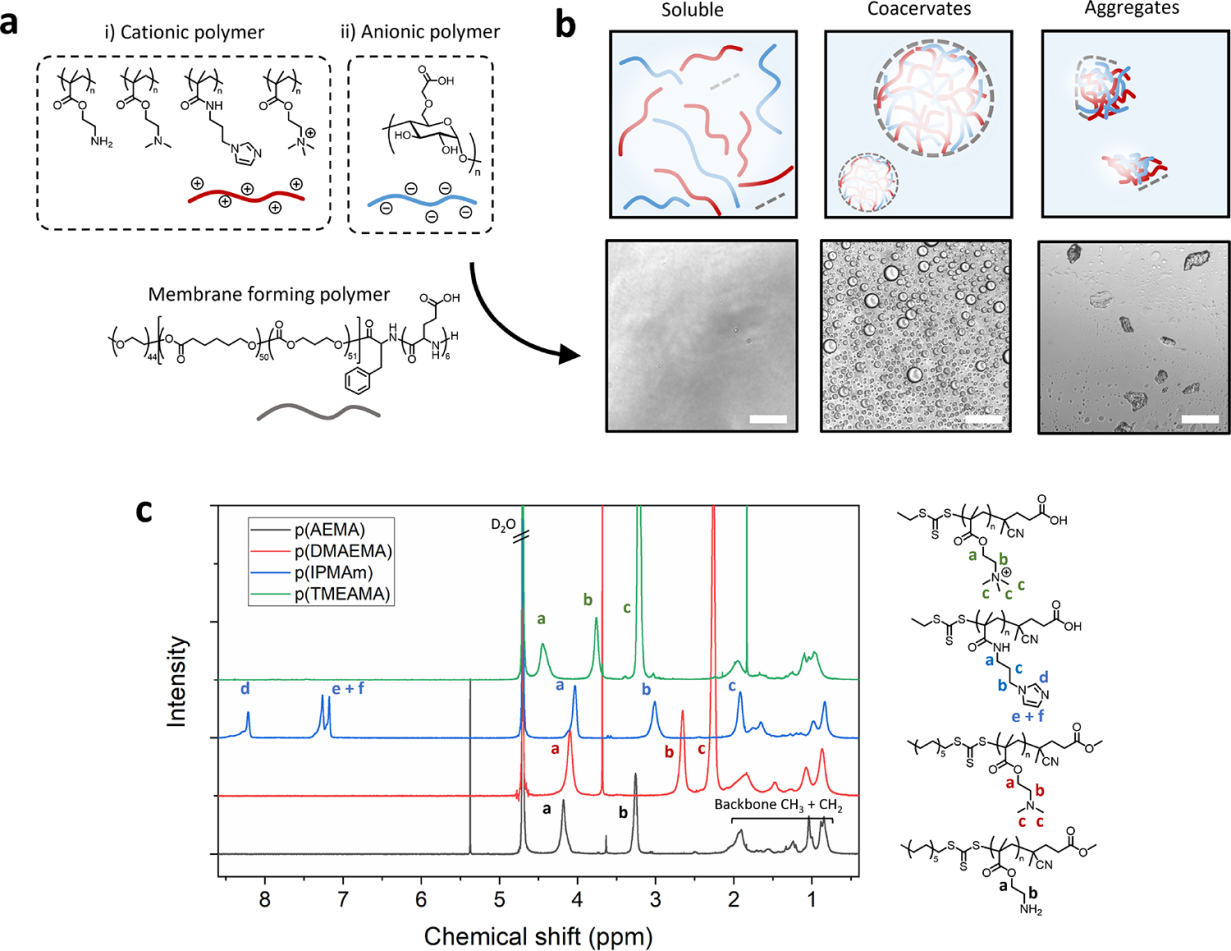
Overview of synthetic polymers used for complex coacervate formation and behavior indicative of liquid–liquid phase separation. (a) Chemical structures of synthesized cationic polymers, p(AEMA), p(DMAEMA), p(IPMAm), p(TMAEMA), carboxymethyl amylose, and membrane forming terpolymer mPEG-p(CL-*g*-TMC)-pGlu. (b) Formation of soluble polymer mixture, complex coacervate cell mimicking droplets, or aggregation of charged polymers. (c) ^1^H NMR spectra of synthesized cationic polymer structures in D_2_O.

All polymerizations were carried out with the same general protocol. The molar ratios of monomer, chain transfer agent, and azoinitiator are shown in the reaction schemes of Figures S2–S5. In a typical example, chain transfer agent methyl 4-cyano-4-(dodecylthiocarbonothioylthio)pentanoate (26.6 mg, 0.064 mmol), 2-(dimethylamino)ethyl methacrylate, DMAEMA (0.5 g, 3.180 mmol), 4,4′-azobis(4-cyanopentanoic acid), ACVA (0.89 mg, 0.0032 mmol), and dioxane (0.176 mL) were added to a vial deoxygenated by bubbling with argon and left to stir in an oil bath at 70 °C. After 24 h, the solution was removed from the oil bath and the polymer precipitated three times in diethyl ether (or hexane depending on the monomer) and dried under vacuum. ^1^H NMR spectra of the polymers (deuterated water D_2_O) are shown in the main text (Figure 1c) and in detail in the Supporting Information (Figures S8–S11).

### Polymer Characterization

^1^H nuclear magnetic resonance (NMR) spectra were conducted on a Bruker Advance 400 MHz spectrometer in either deuterated chloroform (CDCl_3_) or deuterated water (D_2_O). Gel permeation chromatography (GPC) was recorded by using a Shimadzu Prominence-I GPC system. The system was configured with a pLgel-mixed D column and a Shimadzu RID-20A differential refractive index detector. The used eluent was tetrahydrofuran (THF) with a flow rate of 1 mL/min, and polystyrene calibration standards were used. Potentiometric titration of the polymers was performed in a 20 mL vial with a Mettler Toledo pH meter and a general-purpose probe. Solutions of polymer (1 mg/mL) were prepared with deionized water (at room temperature), and 1 M NaOH was added to raise the starting pH to approximately 12. Titrant HCl (0.01 M) was gradually added with micropipettes under gentle stirring; after reaching a stable pH value, the base was again added. The titration curve and the first derivative of the curve were used to determine the equivalence points and the p*K*_a_, taken at half the equivalence points. For *p*(IPMAm), 50/50 ethanol/water was used as solvent due to polymer precipitation above its p*K*_a_ in pure water.^22,23^

### Membranized Complex Coacervate Formation

Polycationic polymers and CM-amylose with a DS of 0.43 were dissolved separately in 1× PBS. Coacervation was induced by the addition of polycation (50 μL) to CM-amylose (50 μL) in molar charge ratios polycation:CM-Am 2:1 while shaking at 1400 rpm. After 5 min, the terpolymer (4 μL, 25 mg/mL in DMSO) was added. The terpolymer, mPEG-p(CL-*g*-TMC)-pGlu, was synthesized according to our previous work^24^ and is briefly described in the Supporting Information. When loading succ-BSA-488, the protein (1 μL, 1 mg/mL) was added directly after coacervation, and then after 5 min, terpolymer (4 μL, 25 mg/mL in DMSO) was added. β-Gal was loaded likewise. Enzyme concentrations for the free proteins were determined using the standard protein absorbance at 280 nm obtained by the NanoDrop (for β-Gal) and by fluorescence measurements for succ-BSA-488 in the supernatant, after centrifuging the coacervate suspension at 500*g* for 5 min. In the case of absorbance measurements, the RAFT CTA trithiocarbonate absorbance was subtracted by using the baseline subtraction function in the NanoDrop software from the supernatant of a control coacervate sample (of the corresponding cation but without enzyme) centrifuged in the same way at the same time. An extinction coefficient of 20.9 for β-Gal was used.

### Brightfield Microscopy

Brightfield microscopy was conducted with a Zeiss AX10 ObserverD1, and the images were analyzed with Fiji (ImageJ). The size distributions of different coacervate formulations were determined by using standard ImageJ functions. The droplets were selected by manual thresholding, and droplets on the edge of the image were excluded from the analysis. A minimum of 80 droplets was analyzed per sample.

### Turbidity

Turbidity assays were performed on membranized coacervate artificial cells with a fixed molar charge ratio of polycation:CM-Am 2:1. Measurements were performed to evaluate the resilience of the coacervate system, formed with different cationic polymers, toward increasing sodium chloride concentration by adding different volumes of 2 M NaCl. Absorbance readings were taken at 500 nm, and turbidity was calculated as 100 – %*T*. The critical concentration was determined by nonlinear curve fitting in OriginLab and taking the inflection point from the fitting equation *y* = A1 + (A2 – A1)/(1 + 10^((LOG*x*0 – *x*) × *p*)). This calculated salt concentration does not take into account ions from other sources (than the added NaCl), and real critical concentrations could therefore be higher. The same measurements were also performed to evaluate the stability of the coacervates to pH changes, in which case the molar charge ratio of polycation:CM-Am was 2:1.

### Confocal Microscopy

Coacervates were imaged with a Leica TCS SP5X (40× objective) confocal laser scanning microscope equipped with a white light laser operating at 50% power; the pinhole was set to 1 Airy Unit. Eighteen-well μ-slides (Ibidi) were used to image coacervate suspensions. For imaging of the Succ-BSA-488, a laser set at 488 nm and emission of 510–550 nm was used. Images were analyzed by using Fiji (ImageJ). Fluorescence intensity profile noise was reduced by using a rectangular fluorescence intensity profile.

### Fluorescence Recovery after Photobleaching (FRAP)

Coacervate droplets were prepared as described above and loaded with 250 nM of Succ-BSA-488. A 100 μL portion of each sample was transferred on a μ-side 18-well glass bottom (Ibidi). FRAP experiments were performed with the FRAP interface available in the Leica LAS software. For imaging, the same settings were used as described above. An initial image was acquired in order to define the region of interest (ROI), 5–10 μm in diameter, within a coacervate. Following, three images of 1024 × 1024 were acquired prior to the bleaching. Subsequently, the ROI was bleached for five iterations of 488 nm, 100% laser power. The recovery was monitored at a 5 s interval. The intensities of the bleached ROI, reference area, a nearby coacervate that was not bleached, and background were extracted from the images with FIJI. Data were normalized by removing the background intensity and dividing by the intensity of the reference area. A first-order exponential equation was fitted using Origin 2020 (OriginLab) from which the immobile fraction, recovery half-life, and *D*_app_ were calculated as reported (fittings shown in Figure S15).^25,26^ The immobile fraction (IM_f_) of the fluorescent protein was calculated from the following eq (eq 1), where *I*_plat._ is the fluorescence intensity at the recovery plateau, *I*_0_ is the bleached fluorescence intensity, and *I*_i_ is the initial fluorescence intensity.
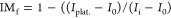
1

The half-time of recovery (τ_1/2_) was calculated from eq 2, where τ is the fluorescence recovery time constant.
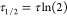
2

The apparent diffusion coefficient (*D*_app_) was calculated following eq 3, an approximation reported in the literature that assumes 2D diffusion, where ω is the radius of the bleached region.
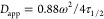
3

### Enzyme Assay

Enzyme activity was evaluated by using a Tecan Spark multimode microplate reader. Coacervates were formed as described above and loaded with β-Gal enzyme. 50 μL of polycation was mixed with 50 μL of CM-Am (both in 10 mM PBS with 1 mM Mg^2+^) at 1500 rpm, followed by the immediate addition of β-Gal (1 μL of 1 mg/mL). After 5 min of mixing, 4 μL of 25 mg/mL terpolymer stock was added. Technical triplicates of enzyme-loaded coacervates were made as described as above. A fraction of the samples were centrifuged for 1 min at 12,000 × *g*, and the supernatant was analyzed with NanoDrop to calculate enzyme encapsulation efficiency (shown in Figure S16). Following this, coacervates containing enzyme were added to the wells of a nonbinding black 96-well microplate with a transparent bottom (Greiner Bio-One), with the 4-MUG substrate preadded to the wells to achieve final substrate concentrations from 10 to 1000 μM. Product formation was monitored for over 30 min, and a measurement was taken every 30 s determining the indoxyl fluorescence (ex. 365 ± 20 nm, em. 445 ± 20 nm) at 37 °C (an example is shown in Figure S17).

A standard curve of 4-methylumbelliferone (in PBS, diluted from DMSO) was used to determine the concentration of the product from the fluorescence values. We observed no significant difference between 4-methylbelliferone standard curve buffer fluorescence values and values obtained with coacervates present. To obtain the Michaelis–Menten plots, the initial rate was calculated from the linear increase in product formation over the first 3 min, and this rate was plotted against substrate concentration. The error bars represent the standard deviations of three repetitions. The Michaelis–Menten curves were fitted with Origin 2020 software, with the kinetic parameters shown in Table S1.

## Results and Discussion

We sought to design a small range of synthetic homopolymers with varying cationic monomers to incorporate into our established membranized coacervate-based protocell platform (as can be seen in Figure 1).^24,27^ While molecular weight,^28^ architecture,^29−31^ and monomer sequence,^32−34^ are known to play a role in polyionic complexation, we targeted a midlength linear homopolymer library in order to minimize the effect of these variables, thereby focusing on cationic substituent structure. RAFT polymerization was employed to target a degree of polymerization of 50 and retain a narrow molecular weight distribution, while also allowing the polymerization of a range of monomer functionalities.^35−37^ The monomers (2-Boc-amino)ethyl methacrylate, 2-(dimethylamino)ethyl methacrylate, [2-(methacryloyloxy)ethyl] trimethylammonium chloride, and 3-(imidazole)propyl methacrylamide were chosen to give primary amine, tertiary amine, quaternary amine, and imidazole tertiary amine functional polymers. Polymerization conditions can be found in the Supporting Information but in general were carried out at 70 °C for 24 h with differing solvents.

Synthesized cationic polymers were characterized by ^1^H NMR as depicted in Figure 1c and show the expected broad polymer peaks associated with the polymer backbone as well as the side-chain functionalities. Number-average molecular weights from NMR were calculated to be between approximately 8.0–11.0 kg/mol for all polymers (Table 1). The Boc-protected primary amine polymer p(Boc-AEMA) had a low dispersity of 1.18, and p(DMAEMA) a dispersity of 1.22, while the quaternized amine polymers p(TMAEMA) and p(IPMAm) could not be characterized with GPC due to solubility issues. The Boc group of the primary amine polymer was deprotected with TFA (Figure S7) to give the water-soluble p(AEMA).

**Table 1:** Synthesis and Characterization Data of the Cationic Polymers, Synthesized Using Reversible Addition–Fragmentation Chain Transfer (RAFT) Polymerization, and Used in This Study to Form Complex Coacervates

sample	[M]_0_/[CTA]_0_	conversion^a^ (%)	*M*_n,theo_^b^ (g/mol)	*M*_n,NMR_ (g/mol)	*Đ* (GPC)^c^	p*K*_*a*_	charge density pH 7.4 (per 1 kDa)
p(AEMA)	50	98^d^	8480	7990	1.18^d^	7.7	5.2
p(DMAEMA)	50	95	7890	9220	1.22	7.6	3.9
p(IPMAm)	50	65	6540	9540		6.9	1.2
p(TMAEMA)	50	98	10,390	11,420			5.8

a Conversion determined by ^1^H NMR spectroscopy.

b *M*_n,theo_ = ([M]_0_/[CTA]_0_ × MW(M)) × (conv. %) + MW(CTA).

c *Đ* determined by GPC analysis in THF.

d Values obtained for the precursor polymer p(Boc-AEMA).

**Figure 2:**
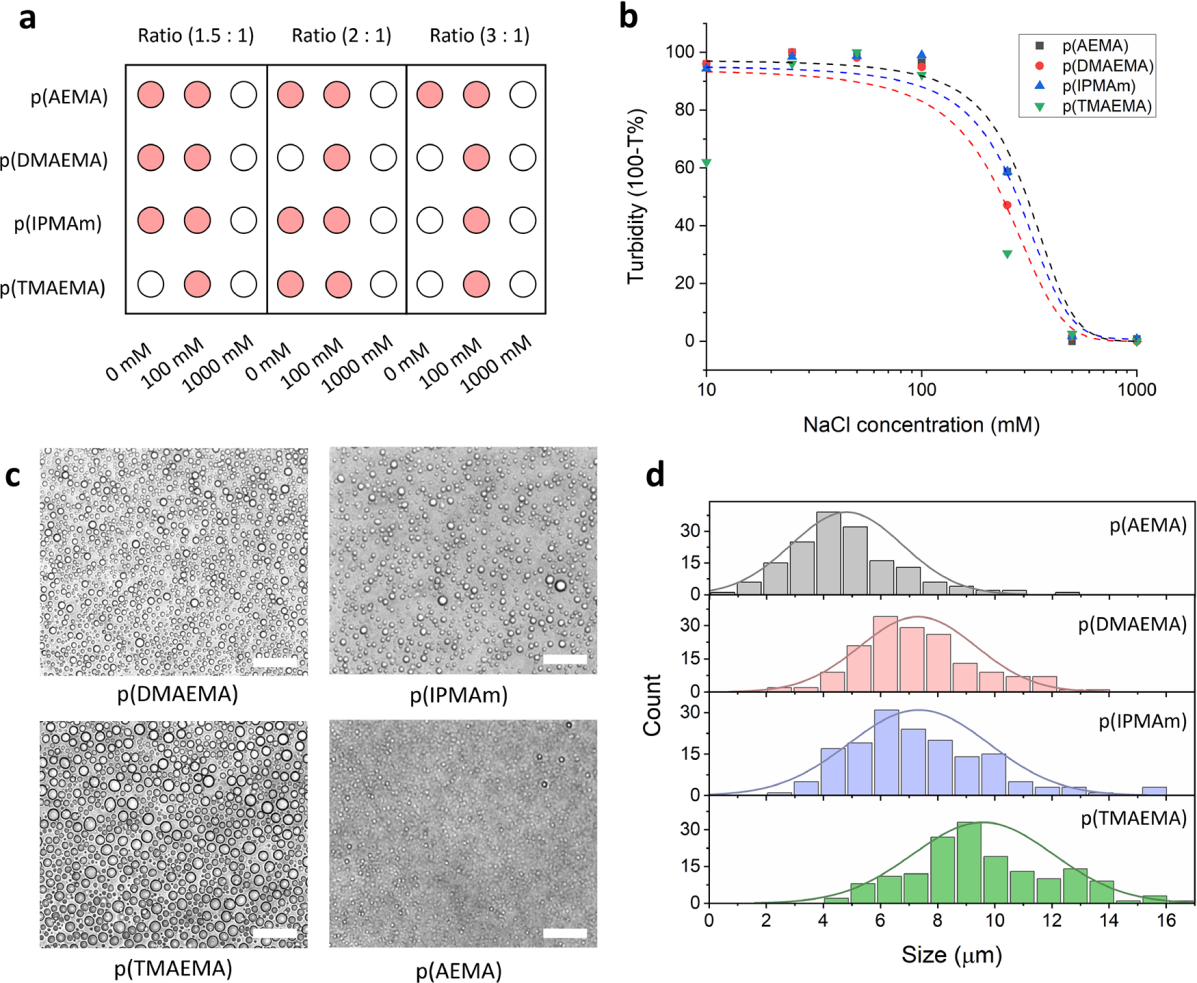
Complex coacervate droplet formation from cationic and anionic polymers. (a) Investigation of charge molar ratio, and salt concentration, on coacervate formation with different polymers (white = soluble, red = coacervate droplets); corresponding microscopy images in the Supporting Information. (b) UV/vis turbidity assay of coacervation with various cationic polymers at a 2:1 charge molar ratio to the anionic polymer (absorbance measurements at 500 nm). (c) Representative brightfield microscopy images of membranized coacervate artificial cells at 2:1 charge ratio and 100 mM NaCl concentration, scale bars 100 μm. (d) Quantification of droplet size (diameter) through microscopy image analysis, *n* > 100 droplets per sample.

In previous work, we formed coacervate artificial cells via the associative segregation of positively charged quaternized amylose (Q-Am) and negatively charged carboxymethyl amylose (CM-Am) in a 2:1 Q-Am:CM-Am molar charge ratio, followed by stabilization with a membrane forming synthetic polymer terpolymer mPEG-p(CL-*g*-TMC)-pGlu. Uptake and concentration of enzymes, nucleic acids, and small molecules in the coacervate interior were affected by the cargo’s extent of negative charge and electrostatic interaction. To investigate the impact of cationic polymer chemical identity on coacervate formation, we first formed coacervates using the same process (replacing Q-Am), at the same neutral pH value of 7.4. The polymer mixtures were characterized by brightfield microscopy to be coacervate forming, to be aggregate forming, or to remain as solutions (representative images shown in Figure 1b). Initially, NaCl concentrations of 0, 100, and 1000 mM were used and overall polymer charge ratios (cationic polymer:CM-Am) of 1.5:1, 2:1, and 3:1 chosen. At an intermediate NaCl concentration, 100 mM, all polymer combinations at all charge ratios tested formed stable coacervate droplets (red circles, Figure 2a). At a polymer ratio of 1.5:1, there was some inconsistent aggregate formation at 0 mM NaCl concentrations. At the highest off-stoichiometric charge ratio 3:1, most combinations at 0 and 1000 mM NaCl remained as polymer solutions.

Further understanding of the impact of the cationic polymer type on coacervation was acquired using turbidity assays, allowing investigation of a wider range of NaCl concentrations. Turbidity was assessed using absorption spectroscopy (at 500 nm) for all four polymer combinations (Figure 2b). Due to the potential of light scattering from aggregates, all samples were checked by microscopy to confirm that turbidity was due to coacervate formation. The critical NaCl concentration for the formed coacervates was found, by nonlinear curve fitting, to be between 180 and 310 mM for all 2:1 charge ratio coacervates. Primary amine coacervates from p(AEMA) had a slightly higher NaCl stability (307.8 ± 41.4 mM) than those formed from the other cationic polymers (pDMAEMA, 236.6 ± 52.0, pIPMAm, 278.3 ± 38.9 mM), with p(TMAEMA) having the lowest NaCl stability of around 200 mM (not calculated, however, due to poor data fit quality). This behavior follows the general expected trend of reduced charge density polymers giving lower salt stability coacervates, apart from the quaternized polymer, which has the lowest salt stability. It is hypothesized that the anomalous behavior of pTMAEMAs is due to existing chloride counterions from the supplied monomer. Spruijt et al. saw similar trends in experimentally measured critical salt concentrations of coacervates made from poly(acrylic acid) with either p(TMAEMA) or p(DMAEMA) or poly(allylamine hydrochloride).^38,39^ In their work, the critical salt concentration also increased from p(TMAEMA) to p(DMAEMA) to primary amine polymer poly(allylamine hydrochloride), although they observed values a factor of two to three times higher than in our case, which is possibly due to the longer-chain polymers used. The average droplet sizes of the coacervates were measured with image analysis in ImageJ and followed a trend similar to that of droplet salt stability; p(AEMA)-based coacervates had an average diameter of 4.81 ± 1.9 μm, p(DMAEMA) 7.31 ± 2.0 μm, p(IPMAm) 7.35 ± 2.5 μm, and p(TMAEMA) 9.59 ± 2.5 μm. These size variations followed the trend of increasing size with reducing charge density, apart from coacervates of p(TMAEMA), which resulted in the largest size droplets.

**Figure 3:**
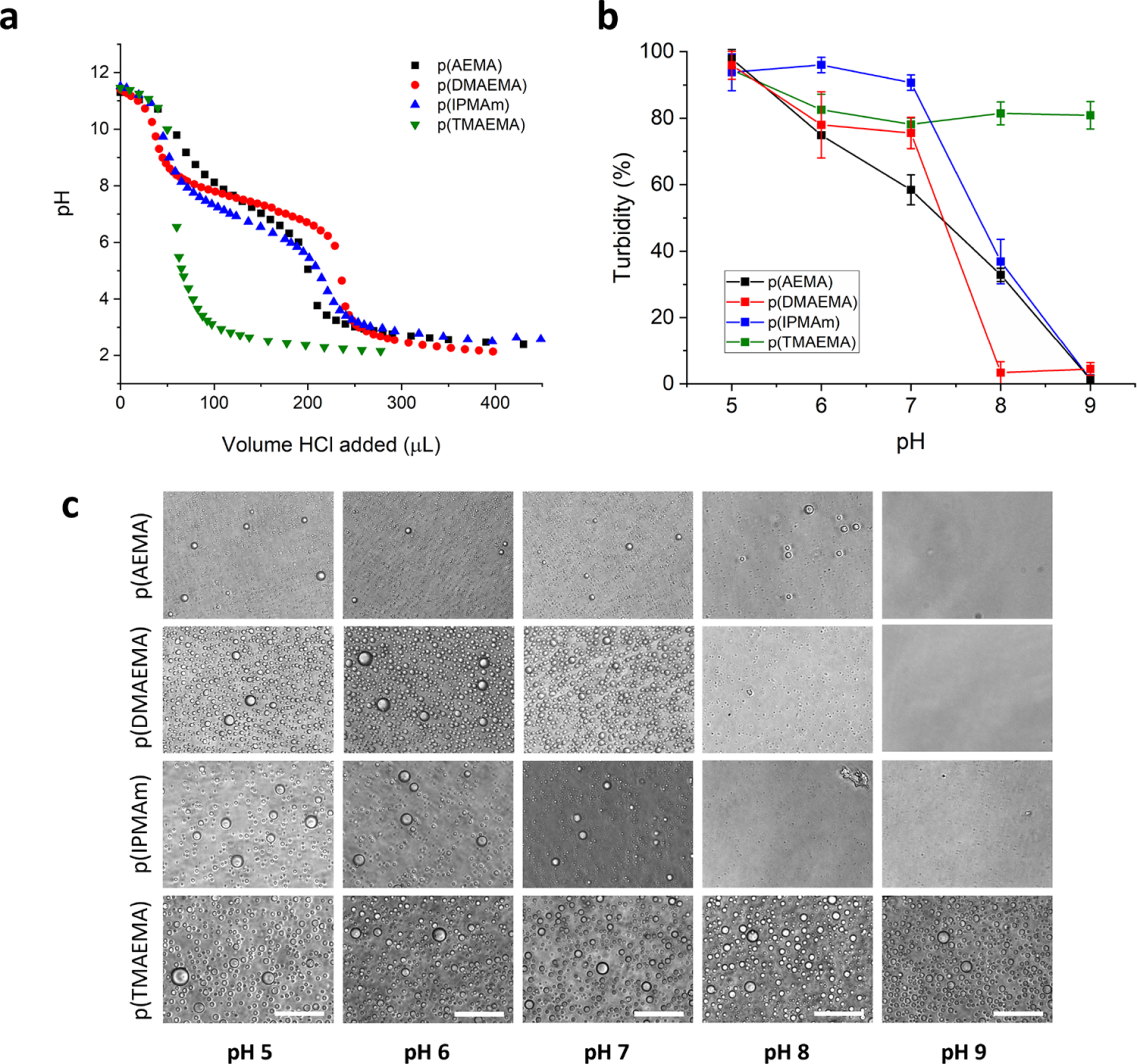
Charge state of cationic polymers and effect of solution pH on droplet formation. (a) pH titration of synthesized amine-containing polymers for assessment of polymer p*K*_a_. (b) Coacervate artificial cell turbidity assays with varying solution pH values (absorbance measurements at 500 nm). (c) optical microscopy images of complex coacervates at varying solution pH values, scale bars 100 μm.

The polymer acid dissociation constants, p*K*_a_’s, were assessed with pH titrations, and the p*K*_a_ was taken as the half equivalence point in the titration curve. Figure 3a shows the pH titration curves of the synthesized cationic polymers, starting from the deprotonated forms. The apparent p*K*_a_ of p(AEMA) was found to be 7.7, and this decreased to 7.6 for p(DMAEMA) and 6.9 for p(IPMAm). Compared to literature values of p*K*_a_’s of ionizable polymers, these results are in the expected ranges: ∼7.6–8.4 for p(AEMA), ∼7.5–7.8 for pDMAEMA, and ∼5.9–6.9 for similar imidazole-containing polymers.^22,40−42^ p(TMAEMA) was included as a control and was not expected to show any buffering capacity due to its quaternary amines. The degree of protonation of the polymers at pH 7.4 can be calculated from the Henderson–Hasselbalch equation and was used to determine the charge density, which is shown in Table 1. Charge density is an important parameter that can affect the functional application of coacervate materials and also phase stability.^20,43,44^ In general, varying charge density affects the coacervate phase formation by changing entropy gained by counterions during coacervation, as well as affecting physical properties by altering the number of points for chain interactions to occur.^45,46^ Still, the situation is sometimes more complicated as besides charge density also hydrophobicity, cation–pi interactions, and chain length play a role.^47,48^

Coacervate turbidity was then measured as a function of pH (at 2:1 polycation:CM-Am ratio), to investigate any pH-triggered coacervate disassembly (Figure 3b). The quaternized polymer formed coacervates that were stable at all pH values tested, which was confirmed by optical microscopy (Figure 3c). The cationic polymers with primary or tertiary amines disassembled to homogeneous solutions at pH 7–8 for p(DMAEMA) and p(IPMAm), and at around pH 8–9 for p(AEMA), based on the microscopy images.

**Figure 4:**
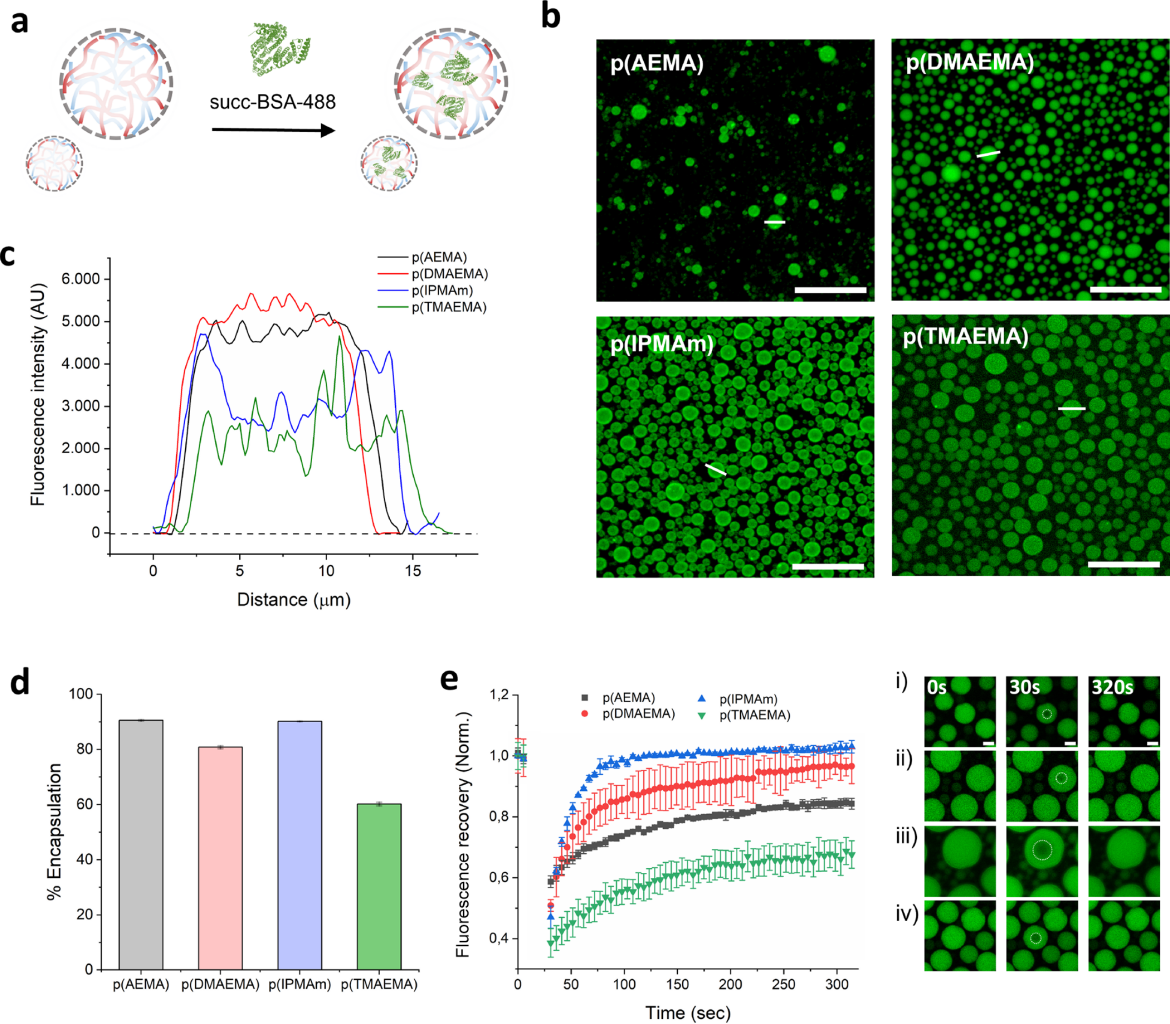
Ability of coacervates to take up proteins and the effect of cationic polymer amine moieties. **a)** Schematic of protein uptake experiments, performed with succinylated bovine serum albumin labeled with Alexfluor488 dye. **b)** Confocal microscopy images showing BSA uptake into artificial cells; scale bars 100 μm. **c)** Fluorescence intensity profiles of droplets showing distribution of protein through the analyzed droplets. **d)** Quantification of BSA uptake into droplets through NanoDrop absorbance measurements of the protein concentration in supernatants after centrifugation. **e)** Fluorescence recovery after photobleaching (FRAP) experiments, showing the effect of cationic polymer on protein diffusion through the crowded droplets.

We then sought to understand the impact of coacervate chemical identity on protein uptake. Previous work has shown that at a 2:1 polycation:CM-Am molar charge ratio, negatively charged proteins are efficiently taken up in the coacervate artificial cell interior.^21^ To verify this for our systems, we studied how the cationic polymer structure affected the recruitment of a model payload protein, fluorescent serum albumin (succinylated-BSA-488), by fluorescence microscopy and fluorescence spectroscopy encapsulation quantification. Confocal microscopy showed efficient uptake of BSA into all four coacervates, as can be seen in Figure 4b. Coacervates made from p(AEMA) and p(DMAEMA) showed particularly homogeneous uptake (visualized by the fluorescence profile plots, Figure 4c), while p(IPMAm) coacervates seemed to have protein accumulation at the droplet exteriors and p(TMAEMA) showed lower green fluorescence intensity. These observations were quantitatively confirmed with fluorescence spectroscopy of the coacervate supernatant after centrifugation of the coacervate phase to give encapsulation efficiency (EE) values (Figure 4d). The EE values ranged from 90.2 and 90.6% for p(IPMAm) and p(AEMA), down to 81.2% for p(DMAEMA)-based coacervates, with the lowest encapsulation values of 60.2% corresponding to the p(TMAEMA) coacervates. These results suggest that monomer structure plays an important role in cargo uptake, due to differing interactions between the polycations and amino acid residues. Interestingly, the p(IPMAm) polycation has the lowest degree of protonation at pH7.4 but still has significant protein sequestration, presumably due to either increased hydrophobic interactions or pi–pi interactions through the imidazole repeating unit.

To investigate further the effect of the polymer structure on any interactions between protein cargo and coacervate material, we performed FRAP experiments on succ-BSA-488 in coacervates (Figure 4e). FRAP is a widely used technique for the biophysical characterization of liquid–liquid phase separated condensates and membraneless organelles. Fitting of the FRAP recovery curves allowed determination of the BSA immobile fraction and the apparent diffusion coefficients *D*_app_ of BSA in membranized coacervate droplets. Regions of interest were chosen as a small fraction of the coacervates to assess internal diffusion of proteins, under the assumption that inter coacervate diffusion would be negligible for proteins of this size (approximately 66 kDa). For p(IPMAm), with the lowest degree of cationic charge, the immobile fraction of BSA was negligible, showing high degree of mobility through the coacervate phase. The values of the BSA immobile fraction obtained for the other coacervates increased with increasing charge density of the polycation. Immobile fractions were 0.133 ± 0.003, 0.368 ± 0.004, and 0.516 ± 0.007, for coacervates comprising p(AEMA), p(DMAEMA), and p(TMAEMA), respectively. The increasing presence of an immobile protein fraction is due to a tight association with static binding sites in the coacervate matrix. Diffusion coefficients were calculated for BSA in the four coacervates from the obtained FRAP curves. *D*_app_ for BSA in these coacervates increased in a trend largely inversely correlated with the protein immobile fraction; the coacervates giving the highest immobile fraction p(TMAEMA) and p(AEMA) gave *D*_app_ of 0.0860 ± 0.0072 and 0.0832 ± 0.0031 μm^2^ s^–1^, respectively. For the condensates giving the lowest immobile fractions p(DMAEMA) and p(IPMAm), the *D*_app_ for these systems was higher: 0.252 ± 0.028 and 0.815 ± 0.057 μm^2^ s^–1^, respectively. Compared to condensates in living cells, the apparent diffusion coefficient of BSA in p(IPMAm) coacervates is similar to GFP in stress granules (*D*_app_ ∼ 1 μm^2^ s^–1^).^49^ The *D*_app_ of proteins in aqueous solution is around an order of magnitude higher.^50^ These noteworthy results indicate that the nature of the cationic groups in complex coacervates and artificial cells plays a very important role in the ability of proteins to freely diffuse through this molecularly crowded environment.

**Figure 5:**
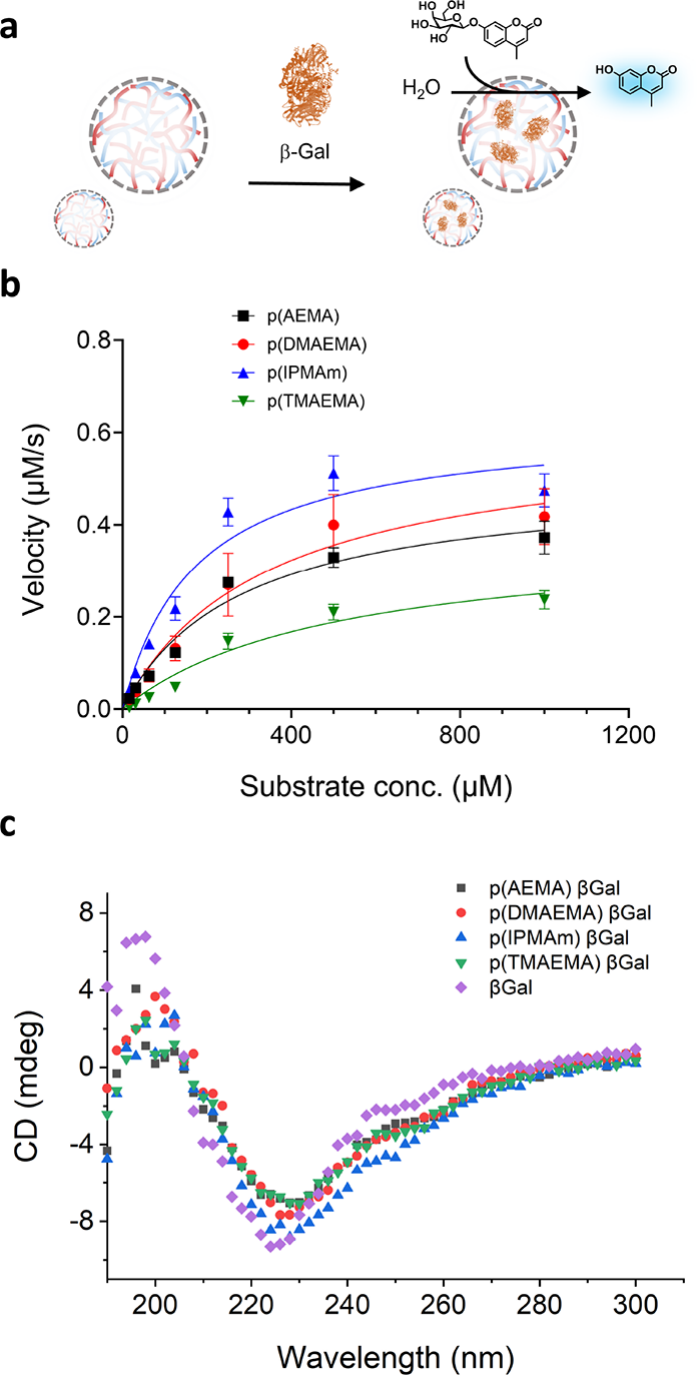
β-Galactosidase enzyme activity inside complex coacervate artificial cells. (a) Schematic showing β-galactosidase uptake into coacervates and subsequent treatment with profluorescent substrate 4-methylumbelliferyl galactopyranoside (4-MUG). (b) Michaelis–Menten kinetic plots of enzymatic reaction after substrate addition, showing differences in enzymatic activity between different coacervate samples. (c) CD spectroscopy profiles of native β-galactosidase showing the protein secondary structure alone and when incorporated in coacervates with different cationic polymer compositions. CD traces show high degrees of secondary structure retention upon protein incorporation in complex coacervates.

Following these interesting insights into the role of polycation structure on protein diffusion in coacervates, we hypothesized that enzyme activity could also be similarly influenced. An enzymatic assay was employed to investigate possible coacervate structure effects on activity using the enzyme β-galactosidase, and its coumarin-based profluorescent substrate (Figure 5a). β-Galactosidase is an 116.3 kDa enzyme assembled into a tetramer, having an isoelectric point of 4.61. The overall negative charge of the protein at neutral pH allows efficient uptake into the coacervate, and after addition of the substrate to the coacervate solution, the fluorescent signal of the cleaved coumarin probe was followed over time at 445 nm for all of the coacervate combinations. Initial rates of reaction were obtained from the initial gradient of the substrate concentration emission slope and compared between coacervate systems. To allow direct comparison of the initial rates of coumarin production, the encapsulation efficiencies and therefore the expected concentration of enzyme inside the coacervate phase were taken into account. NaCl concentrations were kept constant and in excess, so any salt concentration variations were assumed to not affect rates inside coacervated phases. Encapsulation coefficients were 85, 82, 92, and 73% for the four polymer systems studied, p(AEMA), p(DMAEMA), p(IPMAm), and p(TMAEMA), respectively (Figure S16).

The extent that enzymatic activity is affected by inclusion in complex coacervates is still an open question.^51^ Some studies have shown that enzymatic activity is reduced by polymer complexation or inclusion in complex coacervate condensates.^52−54^ On the other hand, in certain situations, enzymatic activity is increased in coacervates.^55,56^ This effect of increasing reaction rates is most obvious in the presence of nonionic polymers.^57,58^ In our experiments, enzymatic production of the fluorescent probe in stabilized coacervate droplets proceeded rapidly, with a fast growth of fluorescent signal before reaching plateau values in emission (Figure S17). Quantification of the initial gradient of the product evolution profile for a range of substrate concentrations affords the Michaelis–Menten curves of enzyme kinetics (Figure 5b). It is clear that the type of coacervate has an impact on enzymatic activity. Values for the Michaelis constant, *K*_M_, and maximum product velocity, *V*_max_, can be seen in Table S1. *K*_M_ values for p(AEMA) and p(DMAEMA) containing coacervates were in the same order as free enzyme (363 μM), with values of 282.1 μM ± 14 and 344.3 μM ± 21, respectively. The *K*_M_ value for the highest charge density polycation, p(TMAEMA) containing coacervates, was slightly higher at 423.0 μM ± 30, indicating a minor amount of inhibition commonly seen for enzymes in macromolecularly crowded environments, while for coacervates from the lowest charge density p(IPMAm), the *K*_M_ value was the lowest at 174.6 μM ± 8, indicating an increased affinity of the enzyme for the substrate, sometimes seen due to upconcentration of the substrate.^56^ It is important to note that apparent enzyme activity could depend on the partitioning of substrate and product; however, in this case, substrate partitioning precleavage is difficult to determine experimentally. The free enzyme in solution has a *K*_M_ value of 363 μM, and the free enzyme with an inhibitor has a *K*_M_ value of 4940 μM.^59^ Overall, the values of *K*_M_ decrease with respect to reducing the charge density of polycation while *V*_max_ decreased for increasing charge density cationic polymers, from 0.622 μM/s down to 0.377 μM/s for p(TMAEMA).

As discussed, the ionic interactions between polycations and enzyme could cause interference due to competition with the active site but could also cause disruption of the β-galactosidase secondary and tertiary structures. Protein secondary structure has been previously observed by FTIR spectroscopy and circular dichroism to not be influenced significantly when the protein itself is adsorbed to (or in) a polyelectrolyte layer of opposite charge.^16,17,60,61^ Having established the successful encapsulation and kinetic parameters of the enzyme β-galactosidase in coacervates, we set out to determine the degree of structural preservation of the protein core by circular dichroism (CD) spectroscopy. Literature data from X-ray crystallography, FTIR, and CD spectroscopies have shown that the structure is dominated by β-sheet conformation with smaller amounts of α-helix structure.^62^ Our data are in line with this, with all samples showing an ellipiticity minimum at ∼220 nm typical for the β-sheet structure. For all coacervate-encapsulated enzyme samples, we observed the minimal influence of the charged polymers on the protein secondary structure but observed a small reduction in the intensity of the signals, indicating a loss in the total energy of folding. This is consistent with a previous report for electrostatically bound enzymes.^63^ We can then consider the active enzymatic site of β-galactosidase and any possible mechanisms for the observed reduction in reaction rate in more densely charged polycation-based coacervates. In β-galactosidase, amino acid residue Glu-537 is key to the galactosidase cleavage mechanism. It is the nucleophile that binds to a galactosyl intermediate during the substitution reaction.^64^ We hypothesize that more highly charge-dense polycations in the coacervate, p(TMAEMA) and p(AEMA), could be more strongly binding to this negatively charged glutamic acid residue and competing with substrate binding. This is kinetic in nature, and the competitive binding assumes reversible changes in the reaction rate. Saburova et al. showed similar results involving the structure and activity of urease when interacting with polyelectrolytes, particularly with the primary amine polymer polyallylamine, compared to secondary amine polymer polydiallyl dimethylammonium chloride. The authors showed that the alpha helical structure of urease when complexed with polymers was retained; however, total enzyme activity was reduced at high polymer concentrations in the case of polyallylamine.^61^

## Conclusions

Despite growing interest in artificial cells and complex coacervates in general, from both a fundamental and an applied aspect, there remains much to uncover about their formation and function. For example, how is their function as a depot or compartment for enzymatic reactions affected by their macromolecular constituents’ microstructure and the overall macroscale characteristics of the complex coacervate? In this study, we synthesized a range of cationic polymers, p(AEMA), p(DMAEMA), p(IPMAm), and p(TMAEMA) via RAFT polymerization. Complex coacervate artificial cells were formed by combining these polymers with an anionic amylose derivative, followed by decoration with a membrane forming a terpolymer, mPEG-p(CL-*g*-TMC)-pGlu, at the droplet interface. Consequently, coacervate formation, stability, protein partitioning, and enzyme function were investigated. The effect of amine functionality and charge density on the outcome of these experiments was considered, and the results show that reduced charge density polycations gave condensates with higher protein mobility and also higher enzyme activity. These results can give insight into how cells have evolved to modulate the activity of enzymes by compartmentalization into condensate phases and could help guide future applications of coacervate artificial cells where high enzyme activity is required, such as sensing, catalysis, or therapeutic applications.

## Supporting Information Available

Experimental details, synthetic procedures, polymer ^1^H NMR spectra, gel permeation chromatography (GPC) traces of synthesized polymers, coacervate brightfield microscopy images, coacervate FRAP fittings, β-gal coacervate encapsulation efficiencies, β-gal coacervate product formation kinetics, and β-gal coacervate Michaelis–Menten parameters (PDF)
