# Supplementary material for: Tuning of Cationic Polymer Functionality in Complex Coacervate Artificial Cells for Optimized Enzyme Activity
Source: Biomacromolecules. 2023 Dec 8;25(1):425–35. doi: 10.1021/acs.biomac.3c01063 (PMC10777345; doi:10.1021/acs.biomac.3c01063)
Supplement: Supplementary file 1 — bm3c01063_si_001.pdf [file bm3c01063_si_001.pdf]

Supporting information

Tuning of cationic polymer functionality in complex coacervate artificial cells for optimised enzyme activity

Alexander B Cook[a]*, Bruno Delgado Gonzalez[b], Jan C M van Hest[ac]*

[a]*Bio-Organic Chemistry, Institute for Complex Molecular Systems, Eindhoven University of Technology, Eindhoven, Netherlands*

[b]*Centro Singular de Investigación en Química Biolóxica e Materiais Moleculares (CIQUS), Departamento de Química Orgánica, Universidade de Santiago de Compostela, Jenaro de la Fuente s/n, 15782 Santiago de Compostela, Spain*

[c]*Biomedical Engineering, Institute for Complex Molecular Systems, Eindhoven University of Technology, Eindhoven, Netherlands*

*Corresponding authors: j.c.m.v.hest@tue.nl; a.b.cook@tue.nl

Contents

Supplementary methods: 4

Synthesis of terpolymer mPEG-p(CL-*g*-TMC)-pGlu 4

p(Boc-AEMA) deprotection 4

CD Spectroscopy 4

Supplementary figures: 5

Figure S1. Reaction scheme for the synthesis of monomer imidazolepropyl methacrylamide (IPMAm) 5

Figure S2. Reaction scheme for the synthesis of Boc protected primary amine functional polymer, p(Boc-AEMA), and subsequent deprotection to give p(AEMA). 5

Figure S3. Reaction scheme for the synthesis of tertiary amine functional polymer, poly(2-(dimethylamino)ethyl methacrylate), p(DMAEMA). 5

Figure S4. Reaction scheme for the synthesis of imidazole functional polymer, poly(imidazolepropyl methacrylamide), p(IPMAm). 5

Figure S5. Reaction scheme for the synthesis of quaternary amine functional polymer poly([2-(methacryloyloxy)ethyl] trimethylammonium chloride), p(TMAEMA). 6

Figure S6. ^{1}H nuclear magnetic resonance spectrum of synthesised monomer imidazolepropyl methacrylamide in deuterated chloroform. 6

Figure S7. ^{1}H nuclear magnetic resonance spectrum of synthesised Boc protected primary amine functional polymer, p(Boc-AEMA), in deuterated chloroform. 6

Figure S8. ^{1}H nuclear magnetic resonance spectrum of deprotected primary amine functional polymer, p(AEMA), in deuterated water. 7

Figure S9. ^{1}H nuclear magnetic resonance spectrum of tertiary amine functional polymer, p(DMAEMA), in deuterated water....7

Figure S10. ^{1}H nuclear magnetic resonance spectrum of imidazole containing polymer, p(IPMAm), in deuterated water....8

Figure S11. ^{1}H nuclear magnetic resonance spectrum of quaternised amine polymer, p(TMAEMA), in deuterated water....8

Figure S12. Gel permeation chromatography GPC trace of synthesised Boc-protected primary amine functional polymer, p(Boc-AEMA), the used eluent was tetrahydrofuran (THF) with a flow rate of 1 mL/min and polystyrene calibration standards. 9

Figure S13. Gel permeation chromatography GPC trace of synthesised tertiary amine functional polymer, p(DMAEMA), the used eluent was tetrahydrofuran (THF) with a flow rate of 1 mL/min and polystyrene calibration standards. 9

Figure S14. Brightfield optical microscopy images of coacervate screening. Membranised coacervate artificial cells formed at varying polycationic polymer:CM-Am ratios, and with different NaCl concentrations. 10

Figure S15. Fluorescence recovery after photobleaching (FRAP) fittings, with fitting model equation and obtained parameter values, of the four coacervate systems studied in this article. Membranised coacervate artificial cells formed at 2:1 polycationic polymer:CM-Am ratio, with loaded fluorescent protein succ-BSA-488. 11

Figure S16. Quantification of β-Gal uptake into coacervates through nanodrop absorbance measurement of the protein concentration in the supernatants after centrifugation. Membranised coacervate artificial cells were formed at 2:1 polycationic polymer:CM-Am ratio...........................12

Figure S17. Enzyme activity inside complex coacervate artificial cells, as demonstrated with pro-fluorescent substrate probe 4-methylumbelliferyl galactopyranoside (4-MUG), at 250 µM final concentration. Kinetic sampling of enzymatic reaction after substrate addition, showing differences in enzymatic activity between different coacervate samples. 12

Table S1. Michaelis–Menten analysis parameters for β-galactosidase localised inside coacervates of varying cationic polymer composition, derived from curve fitting with Origin software. Reactions were performed in PBS (pH 7.4) at 37 °C. 13

References..13

Supplementary methods:

Synthesis of terpolymer mPEG-p(CL-*g*-TMC)-pGlu

The coacervate stabilising membrane terpolymer was synthesised as previously reported by our group.[1,2] Briefly, ε-caprolactone and trimethylene carbonate were polymerised via ring-opening polymerisation initiated by poly(ethylene glycol) monomethyl ether in the presence of methanesulfonic acid catalyst. The terminal alcohol of this polymer was then transformed to a primary amine *via* a Steglich esterification with Boc-L-Phe-OH, and the deprotection of the amino acid Boc group with trifluoroacetic acid (TFA). Lastly, a poly(L-glutamic acid) block was polymerised from this terminal amine by the ring-opening polymerization of N-carboxyanhydride γ-benzyl l-glutamate, followed by hydrogenation. At each step, the polymer identity and successful modification was confirmed by both GPC and ^{1}H NMR spectroscopy.

p(Boc-AEMA) deprotection

The Boc-protected primary amine polymer p(Boc-AEMA) was deprotected under acidic conditions. TFA in methanol was added to a solution of the polymer, the mixture was then heated to 40 °C for 4 hours. After deprotection the integrity of the polymer was confirmed with ^{1}H NMR spectroscopy, following the disappearance of the Boc-derived methyl peaks at a chemical shift of around 1.55 ppm, while the other peaks remaining unchanged.

CD Spectroscopy

Circular dichroism spectroscopy measurements were recorded on a Jasco J-815 between 300 and 190 nm (scanning speeds of 50 nm/min, 2 s digital Integration Time, 1 nm bandwidth, and a data pitch of 2 nm). All samples were measured using a 5 mm quartz cuvette. Enzyme concentrations were 0.5 mg/mL, and when combined with coacervates, the coacervate final concentration varied to obtain sufficient signal-to-noise ratio, and to keep HT values below 650 V. CD spectra are an average of three accumulated measurements and are background subtracted.

Supplementary figures:

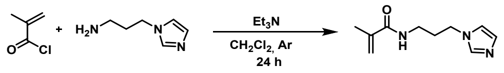

Figure S1. *Reaction scheme for the synthesis of monomer imidazolepropyl methacrylamide (IPMAm)*

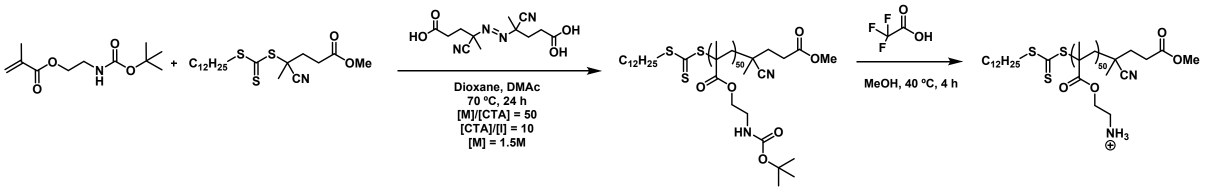

Figure S2. *Reaction scheme for the synthesis of Boc protected primary amine functional polymer, p(Boc-AEMA), and subsequent deprotection to give p(AEMA).*

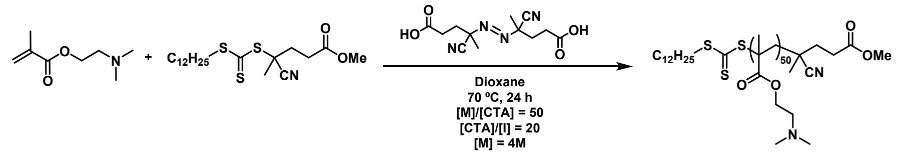

Figure S3. *Reaction scheme for the synthesis of tertiary amine functional polymer, poly(2-(dimethylamino)ethyl methacrylate), p(DMAEMA).*

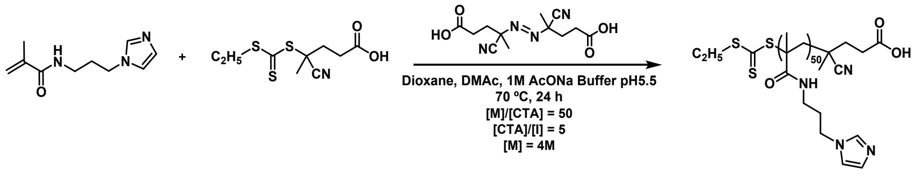

Figure S4. *Reaction scheme for the synthesis of imidazole functional polymer, poly(imidazolepropyl methacrylamide), p(IPMAm).*

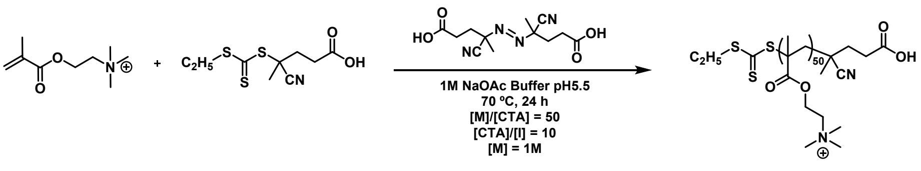

Figure S5. *Reaction scheme for the synthesis of quaternary amine functional polymer poly([2-(methacryloyloxy)ethyl] trimethylammonium chloride), p(TMAEMA).*

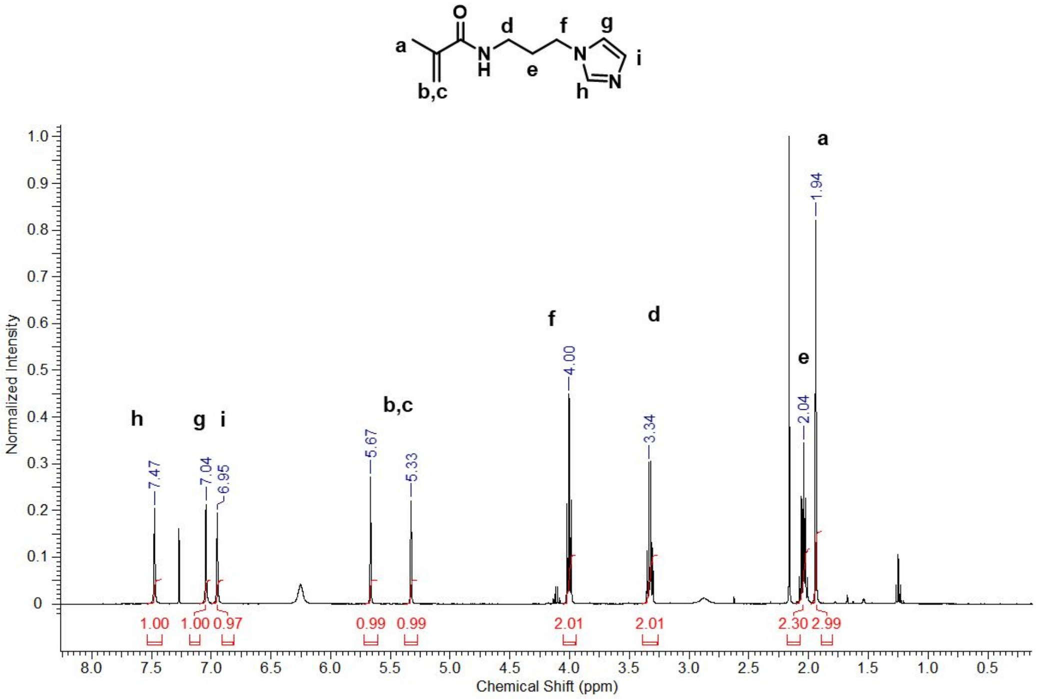

Figure S6. *^{1}H nuclear magnetic resonance spectrum of synthesised monomer imidazolepropyl methacrylamide in deuterated chloroform.*

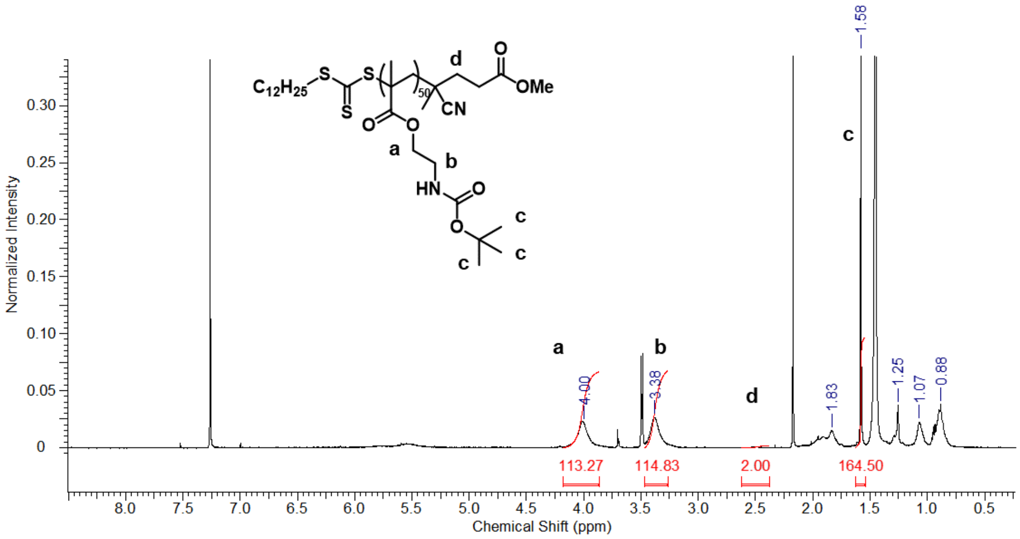

Figure S7. *^{1}H nuclear magnetic resonance spectrum of synthesised Boc protected primary amine functional polymer, p(Boc-AEMA), in deuterated chloroform.*

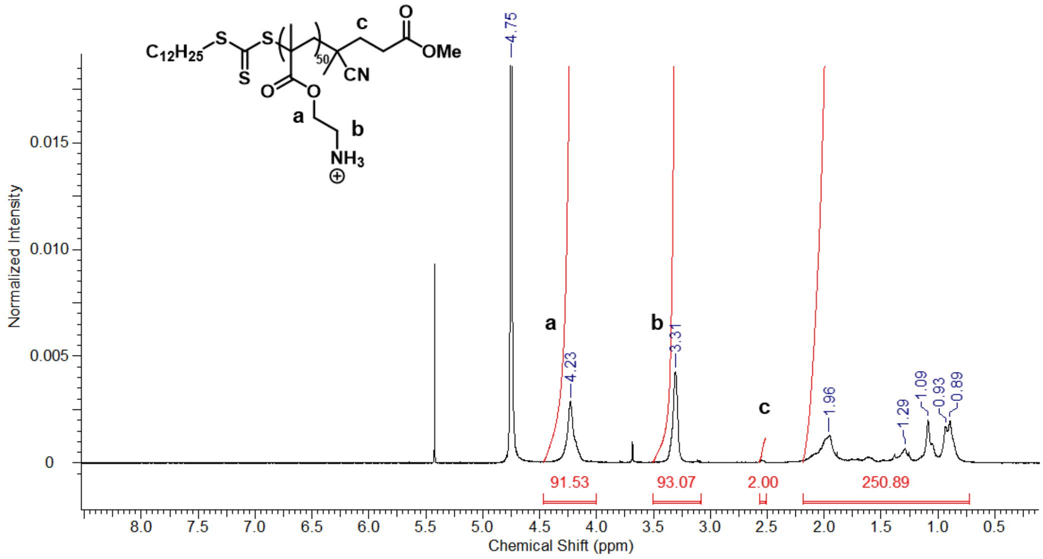

Figure S8. *^{1}H nuclear magnetic resonance spectrum of deprotected primary amine functional polymer, p(AEMA), in deuterated water.*

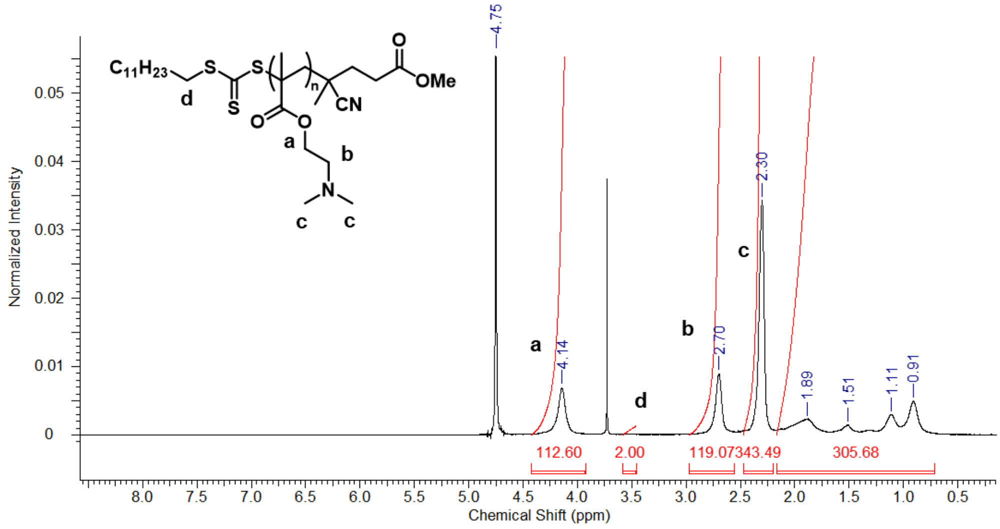

Figure S9. *^{1}H nuclear magnetic resonance spectrum of tertiary amine functional polymer, p(DMAEMA), in deuterated water.*

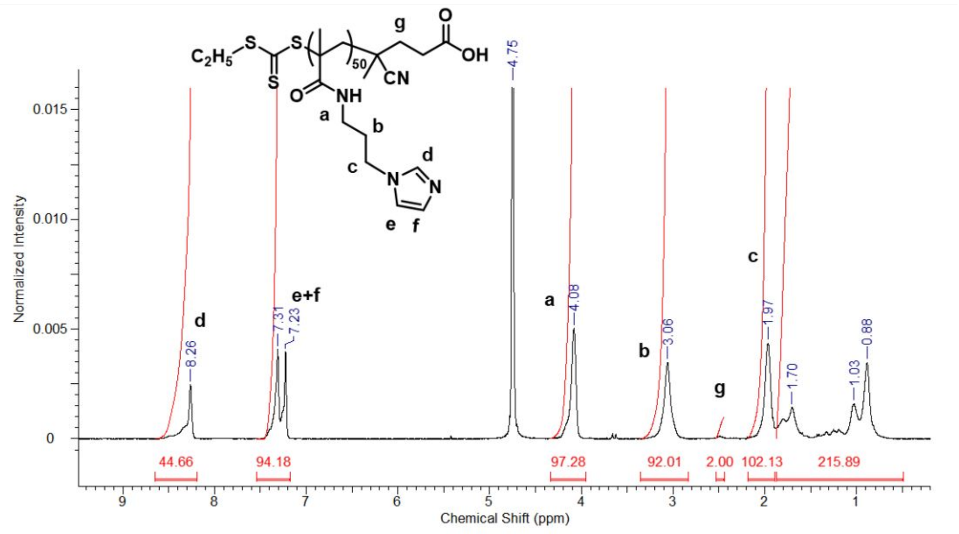

Figure S10. *^{1}H nuclear magnetic resonance spectrum of imidazole containing polymer, p(IPMAm), in deuterated water.*

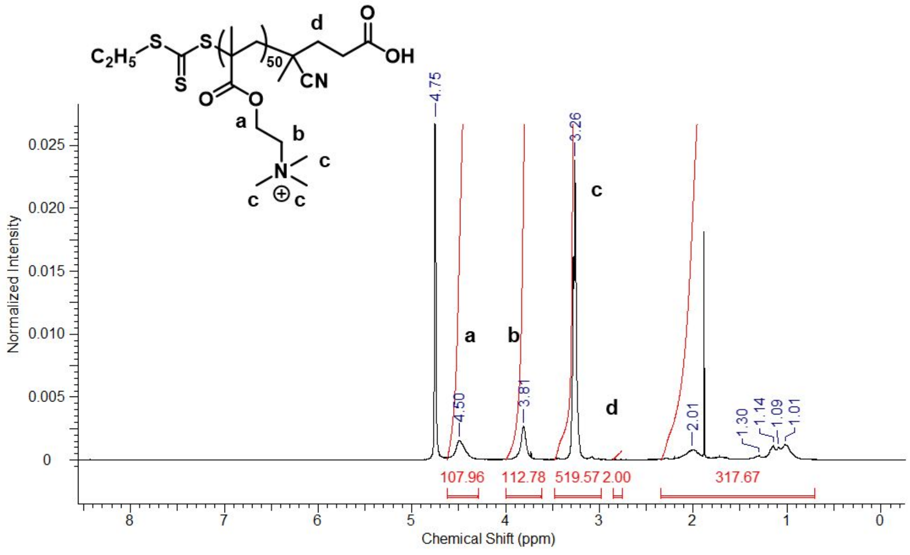

Figure S11. *^{1}H nuclear magnetic resonance spectrum of quaternised amine polymer, p(TMAEMA), in deuterated water.*

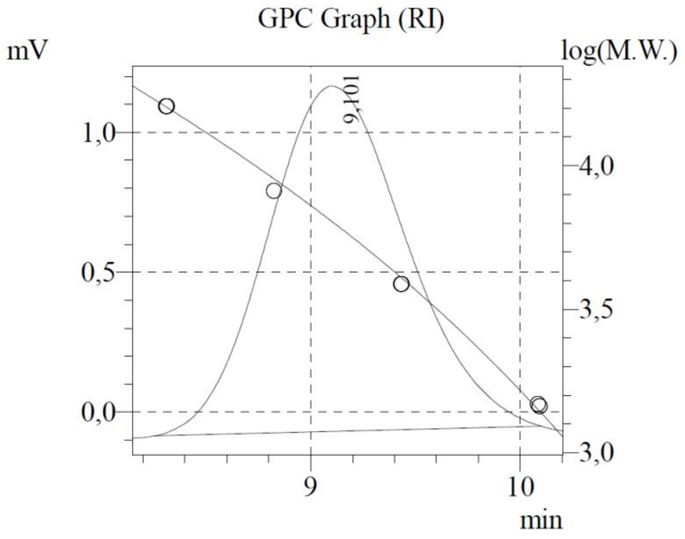

Figure S12. *Gel permeation chromatography GPC trace of synthesised Boc-protected primary amine functional polymer, p(Boc-AEMA), the used eluent was tetrahydrofuran (THF) with a flow rate of 1 mL/min and polystyrene calibration standards.*

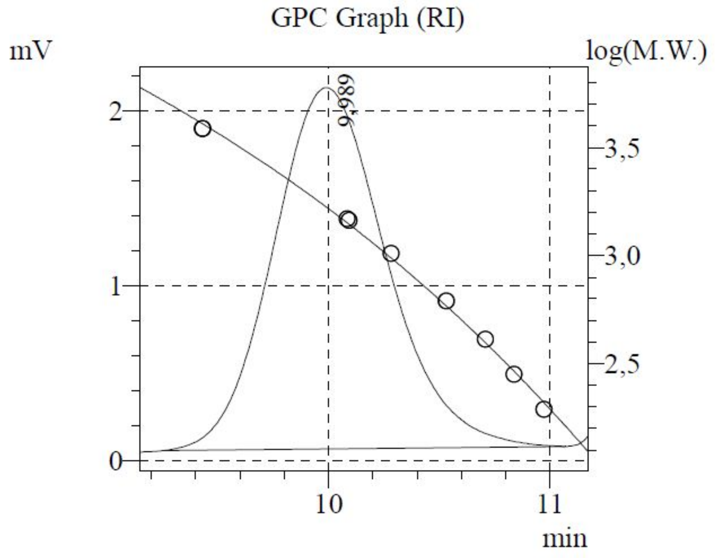

Figure S13. *Gel permeation chromatography GPC trace of synthesised tertiary amine functional polymer, p(DMAEMA), the used eluent was tetrahydrofuran (THF) with a flow rate of 1 mL/min and polystyrene calibration standards.*

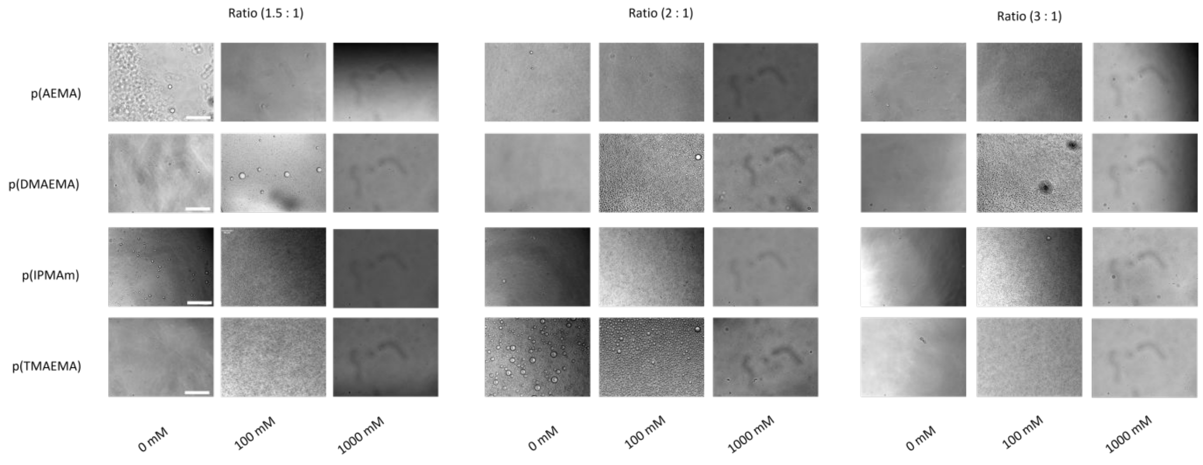

Figure S14. *Brightfield optical microscopy images of coacervate screening. Membranised coacervate artificial cells formed at varying polycationic polymer:CM-Am ratios, and with different NaCl concentrations. Images same dimensions, scale bars 100 µm.*

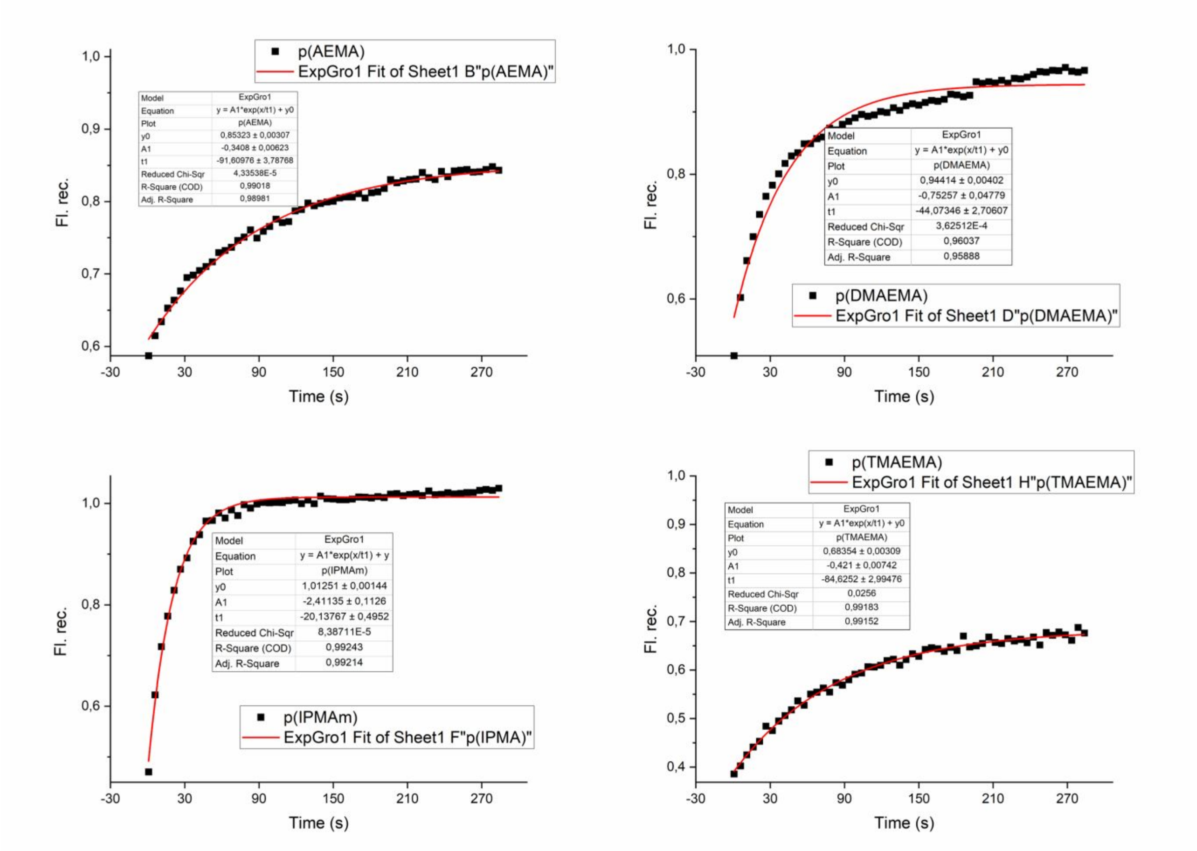

Figure S15. *Fluorescence recovery after photobleaching (FRAP) fittings, with fitting model equation and obtained parameter values, of the four coacervate systems studied in this article. Membranised coacervate artificial cells formed at 2:1 polycationic polymer:CM-Am ratio, with loaded fluorescent protein succ-BSA-488.*

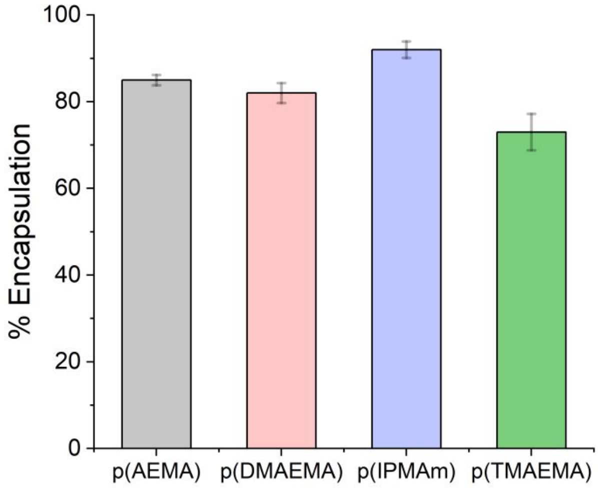

Figure S16. *Quantification of β-Gal uptake into coacervates through nanodrop absorbance measurement of the protein concentration in the supernatants after centrifugation. Membranised coacervate artificial cells were formed at 2:1 polycationic polymer:CM-Am ratio.*

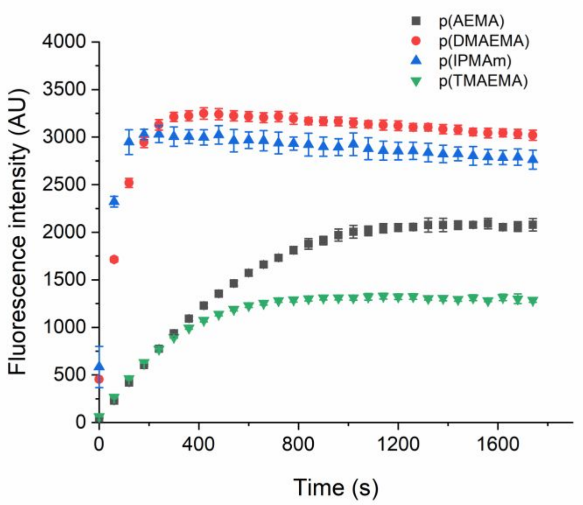

Figure S17. *Enzyme activity inside complex coacervate artificial cells, as demonstrated with pro-fluorescent substrate probe 4-methylumbelliferyl galactopyranoside (4-MUG), at 250 µM final concentration. Kinetic sampling of enzymatic reaction after substrate addition, showing differences in enzymatic activity between different coacervate samples.*

Table S1. *Michaelis–Menten analysis parameters for β-galactosidase localised inside coacervates of varying cationic polymer composition, derived from curve fitting with Origin software. Reactions were performed in PBS (pH 7.4) at 37 °C.*

β-Gal sample	K_M (μM)	V_{max} (μM/s)
p(AEMA)	282.1 ± 14	0.498 ± 0.05
p(DMAEMA)	344.3 ± 21	0.600 ± 0.05
p(IPMAm)	174.6 ± 8	0.622 ± 0.10
p(TMAEMA)	423.0 ± 30	0.377 ± 0.08
β-Gal bulk[3]	363.0	-
β-Gal bulk (+inhib.)[3]	4940	-

References

(1) Mason, A. F.; Yewdall, N. A.; Welzen, P. L. W.; Shao, J.; van Stevendaal, M.; van Hest, J. C. M.; Williams, D. S.; Abdelmohsen, L. K. E. A. Mimicking Cellular Compartmentalization in a Hierarchical Protocell through Spontaneous Spatial Organization. *ACS Cent. Sci.* **2019**, *5* (8), 1360–1365. https://doi.org/10.1021/acscentsci.9b00345.

(2) Mason, A. F.; Buddingh', B. C.; Williams, D. S.; van Hest, J. C. M. Hierarchical Self-Assembly of a Copolymer-Stabilized Coacervate Protocell. *J. Am. Chem. Soc.* **2017**, *139* (48), 17309–17312. https://doi.org/10.1021/jacs.7b10846.

(3) Martino, S.; Tiribuzi, R.; Tortori, A.; Conti, D.; Visigalli, I.; Lattanzi, A.; Biffi, A.; Gritti, A.; Orlacchio, A. Specific Determination of β-Galactocerebrosidase Activity via Competitive Inhibition of β-Galactosidase. *Clin. Chem.* **2009**, *55* (3), 541–548. https://doi.org/10.1373/clinchem.2008.115873.
